# Complete mitochondrial genomes of the highly invasive ant *Paratrechina longicornis* Latreille (Hymenoptera: Formicidae)

**DOI:** 10.1080/23802359.2026.2760060

**Published:** 2026-11-07

**Authors:** Jachoon Koo, Na-Ri Shin, Soo-Jung Suh

**Affiliations:** ^a^ Division of Science Education and Institute of Fusion Science, Jeonbuk National University, Jeonju, South Korea; ^b^ Plant Quarantine Technology Center, Animal and Plant Quarantine Agency (APQA), Gimcheon, South Korea

**Keywords:** Invasive ant, longhorn crazy ant, mitogenome, molecular phylogeny

## Abstract

*Paratrechina longicornis* (Latreille), widely transported across subtropical and tropical regions, is frequently detected during plant quarantine inspections in South Korea and commonly found in port areas. It thrives in both disturbed and undisturbed habitats as a general scavenger, with nests typically established in dry litter, mulch or beneath ground objects. The specimens were collected in Vietnam through the efforts of an international joint research team. The complete mitogenome of *P. longicornis* was sequenced, assembled and annotated. The mitogenome of *P. longicornis* is a circular DNA molecule of 19,102 bp with an A + T content of 80.87%, comprising 13 protein-coding genes, 22 transfer RNA genes and 2 ribosomal RNA genes. Phylogenetic analysis showed that *P. longicornis* clustered with *Acropyga*, *Anoplolepsis*, *Formica*, *Lasius*, *Nylanderia* and *Camponotus* species in the well-supported subfamily Formicinae.

## Introduction

The longhorn crazy ant, *Paratrechina longicornis* (Latreille, 1802) (Hymenoptera: Formicidae), is a small formicine ant species notable for its rapid and erratic foraging movements, which is the basis for its common name ‘crazy ant’ (Wetterer 2008). Originally native to tropical Asia, *P. longicornis* has successfully expanded its range to tropical and subtropical regions worldwide, establishing itself as one of the most widespread invasive ants (Tseng et al. 2023; AntWiki 2026). Morphologically distinguished by elongated antennae, legs and dense setae, this species shows ecological adaptability by nesting across diverse habitats including soil, leaf litter, decaying wood and urban structures (Nickerson and Barbara 2000; Wetterer 2008). Its opportunistic feeding behavior and ability to form extensive foraging trails contribute to its success in colonizing new environments. In particular, the global movement of agricultural commodities, including timber and horticultural products, has increased the risk of unintentional ant dispersal across borders (Schifani et al. 2024). In South Korea, *P. longicornis* has been repeatedly detected in imported plants and ports of entry, highlighting its quarantine importance (Suh and Tasen 2024; Suh et al. 2025). Due to its global distribution, ecological plasticity, and potential to disrupt native ant communities, *P. longicornis* has become an important model organism for studies on invasion biology, urban ecology and species interactions. Mitochondrial genomes have been widely applied in phylogenetic and evolutionary studies of insects, including ants, which serve as key information for understanding social behavior and ecological adaptation.

In this study, we report the mitogenomic sequences of *P. longicornis* and conduct mitogenome phylogenetic analyses based on 13 protein-coding genes, contributing to the broader understanding of ant mitochondrial genomics and invasive species management. Furthermore, the mitochondrial genome sequence provides a useful reference for developing molecular diagnostic markers that can facilitate rapid detection and identification of this invasive species during quarantine inspections.

## Materials and methods

Specimens identified as *Paratrechina longicornis* were collected from Sala Park, Ho Chi Minh City, Vietnam (10°46’18.6“N 106°43’28.5“E) on May 17, 2026 by an international research team through visual inspection and baiting traps (Figure 1). The morphological characteristics of the longhorn crazy ant are as follows: body length 2.3–3.0 mm; body dark brown to black; head elongated oval in shape; antenna 12-segmented, with the scape extending well beyond the posterior margin of the head by approximately two-fifths of its length; eyes elliptical, strongly convex, and placed close to the posterior border of the head; one node between the propodeum and the gaster; legs slender and long (LaPolla and Fisher 2014). A specimen was deposited at the Collection of Plant Quarantine Technology Center, South Korea (https://www.qia.go.kr, Soo-Jung Suh, suhsj97@korea.kr) under the voucher number APQA-20260634-1. The photograph of the specimen in habitus view was taken using a Leica M165C microscope with a Dhyana 400DC-Tucsen camera.

**Figure 1.**
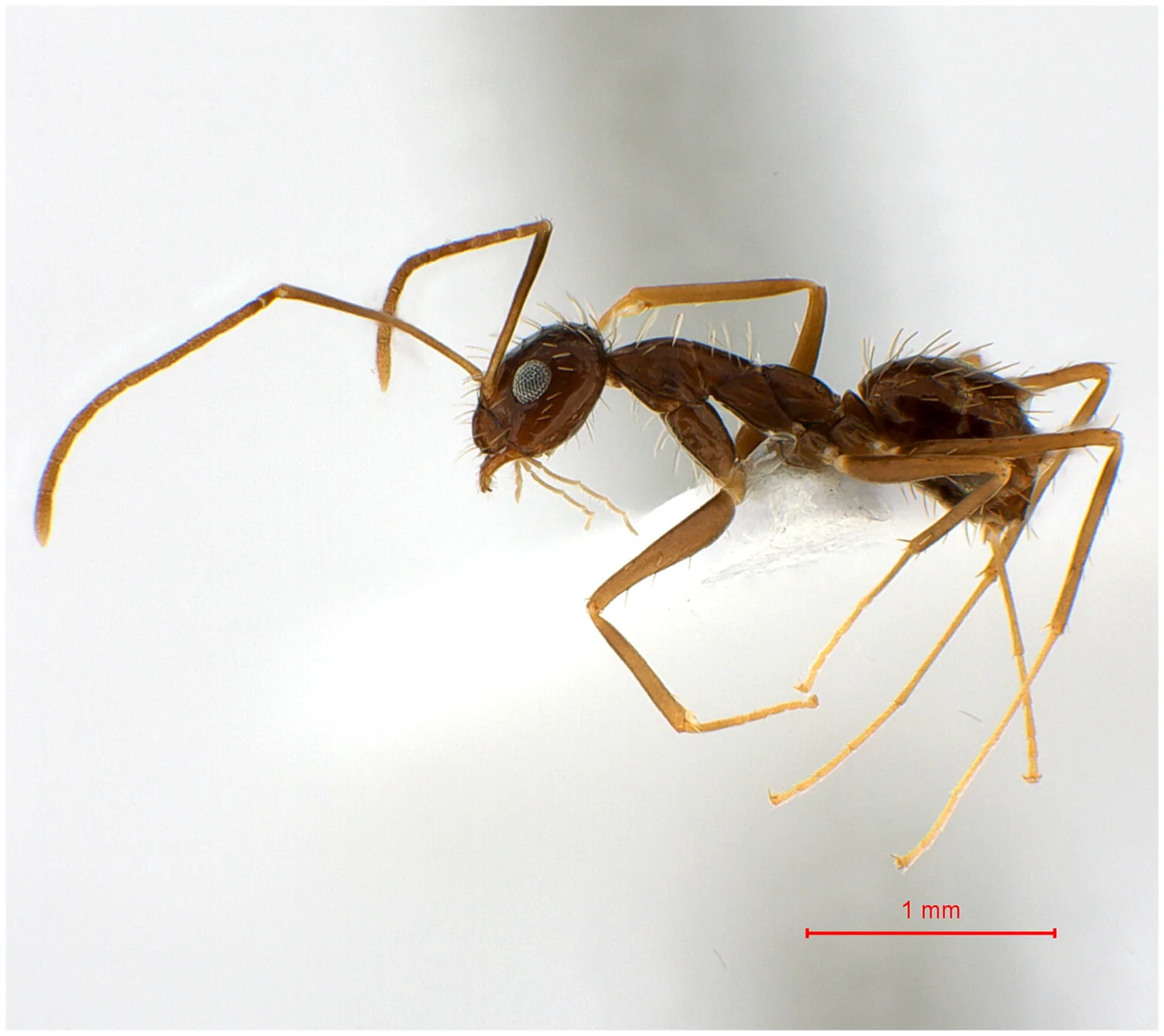
Habitus image of *Paratrechina longicornis* (Latreille). The image was captured using a Leica M165C microscope with a Dhyana 400DC-Tucsen camera by Soo-Jung Suh on August 11 2026. Scale bar: 1 mm.

Genomic DNA was extracted from worker *P. longicornis* using the DNeasy Blood & Tissue Kit (Qiagen, Germany) following the manufacturer’s protocol. DNA integrity was evaluated by agarose gel electrophoresis and using a TapeStation 4200 system (Agilent Technologies, CA). The qualified DNA sample was submitted to PHYZEN (Seongnam, South Korea) for library construction and sequencing. Library preparation was conducted using the TruSeq Nano DNA Library Preparation Kit (Illumina, CA) by manufacturer’s instructions. Paired-end sequencing was performed on an Illumina NovaSeq 6000 platform. Sequencing depth and coverage statistics are shown in Figure S1. A total of 3,686,151,298 bases were generated from 24,411,598 read-pairs. Raw sequencing reads were processed to remove adapter sequences and low-quality bases using Trimmomatic v0.41 (Bolger et al. 2014). The mitochondrial genome was then assembled de novo using GetOrganelle v1.7.7.1 (Jin et al. 2020). The preliminary assembly was further refined by paired-end read mapping with CLC Assembly Cell v4.21 (QIAGEN Digital Insights, Denmark) to correct potential errors and fill remaining gaps (QIAGEN software manual). The circularity and completeness of the assembled mitochondrial genome were subsequently confirmed. Genome annotation was performed using MITOS v2.1.10 (Donath et al. 2019), followed by manual inspection and refinement in the Artemis annotation tool (Rutherford et al. 2000), with additional verification through NCBI BLASTN searches. Finally, a circular map of the mitochondrial genome was generated using the Proksee (Grant et al. 2023).

For phylogenetic reconstruction, mitochondrial genomes from 30 species of Hymenoptera species – including one newly sequenced species and 29 species from GenBank – were analyzed with emphasis on Formicinae, supplemented by representatives of other subfamilies to provide comparative context, and using *Vespula rufa* (Linnaeus) as the outgroup. Phylogenetic analyses were conducted on concatenated nucleotide sequences of 13 mitochondrial protein-coding genes (PCGs), which were extracted, aligned, and managed in PhyloSuite (Zhao et al. 2025), individually aligned with MAFFT v7.505 (Katoh and Standley 2013) using the ‘–auto’ strategy and codon mode, and trimmed with trimAl v1.2 (Capella-Gutiérrez et al. 2009). The resulting alignments were concatenated into a single dataset, with partitioning schemes and substitution models selected by ModelFinder (Kalyaanamoorthy et al. 2017) under the Bayesian Information Criterion (BIC). Maximum likelihood (ML) phylogenetic trees were inferred using IQ-TREE (Nguyen et al. 2015) and branch support was assessed using 20,000 ultrafast bootstrap replicates (Hoang et al. 2018).

## Results

The complete mitochondrial genome sequence of *P. longicornis* is a circular DNA molecule of 19,102 bp (GenBank accession no. PZ830927) comprising 37 genes typical of ant mitogenomes (Figure 2). These included 13 protein-coding genes (PCGs), 22 transfer RNA genes (tRNA) and 2 ribosomal RNA (rRNA) genes, consistent with previously reported ant mitogenomes. Out of the total 13 PCGs, 4 PCGs (ATP6, COX3, CYTB and ND4) started with ATG codon. The remaining genes had the following initiation codons: ND3 and ND6 (ATA); ATP8, COX1, COX2, ND1, ND2, ND4L and ND5 (ATT). Also, all PCGs utilized canonical stop codons, such as TAA and TAG. The overall nucleotide composition of the mitogenome was 39.03% adenine (A), 41.84% thymine (T), 5.58% guanine (G) and 13.55% cytosine (C), indicating a marked A + T bias. The total A + T content was 80.87%. In this species, both AT-skew (−0.035) and GC-skew (−0.417) showed negative values, reflecting a compositional bias toward thymine and cytosine.

**Figure 2.**
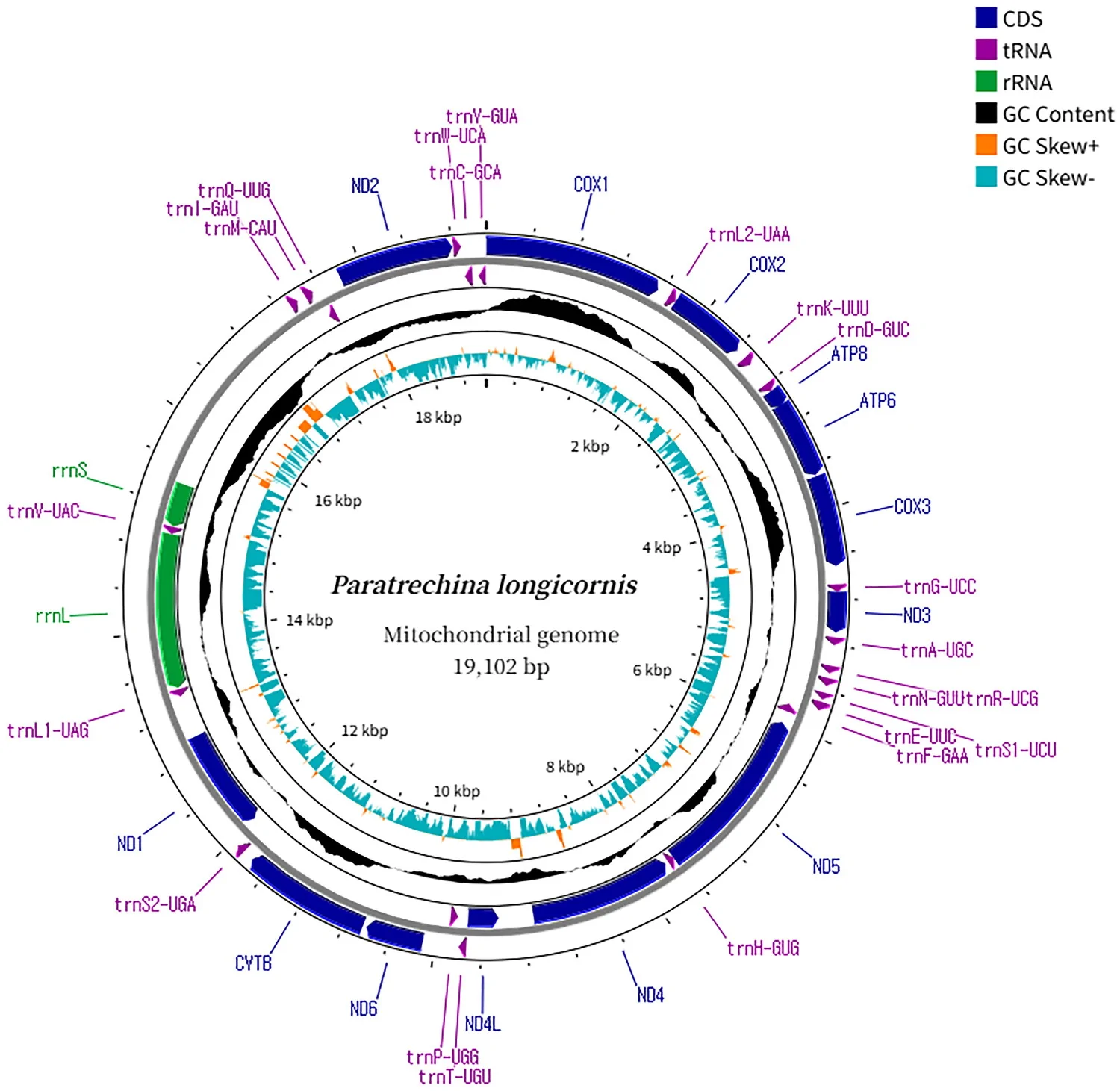
Circular-mapping mitochondrial genome of *Paratrechina longicornis*. Color codes of different types of genes are shown on the legends in the map. The black bar graphs on the inner circle indicate the GC contents, which shows the deviation from the average GC content. The black bar graph on the inner circle indicates GC content, plotted as deviation from the genome-wide mean (19.13%). GC skew is plotted on the innermost circle and calculated as [(G-C)/(G + C)] in 50-bp sliding windows with a 1-bp step size.

A ML phylogenetic tree was constructed using concatenated nucleotide sequences of 13 mitochondrial PCGs from 29 Formicidae species, with one Vespidae species serving as the outgroup. In the resulting tree topology, each subfamily (Formicinae, Myrmicinae, Pseudomyrmecinae, Dolichoderinae, and Ponerinae) formed a distinct monophyletic clade with strong bootstrap support. Within the subfamily Formicinae, *P. longicornis* clustered with *Nylanderia flavipes* (bootstrap support: 100%) and *Lasius spathepus* (bootstrap support: 98%), forming a well-supported clade corresponding to the tribe Lasiini (Figure 3). This branching topology shows a high degree of consistency with previously established phylogenies combining molecular and morphological traits (Ward et al. 2016; Boudinot et al. 2022).

**Figure 3.**
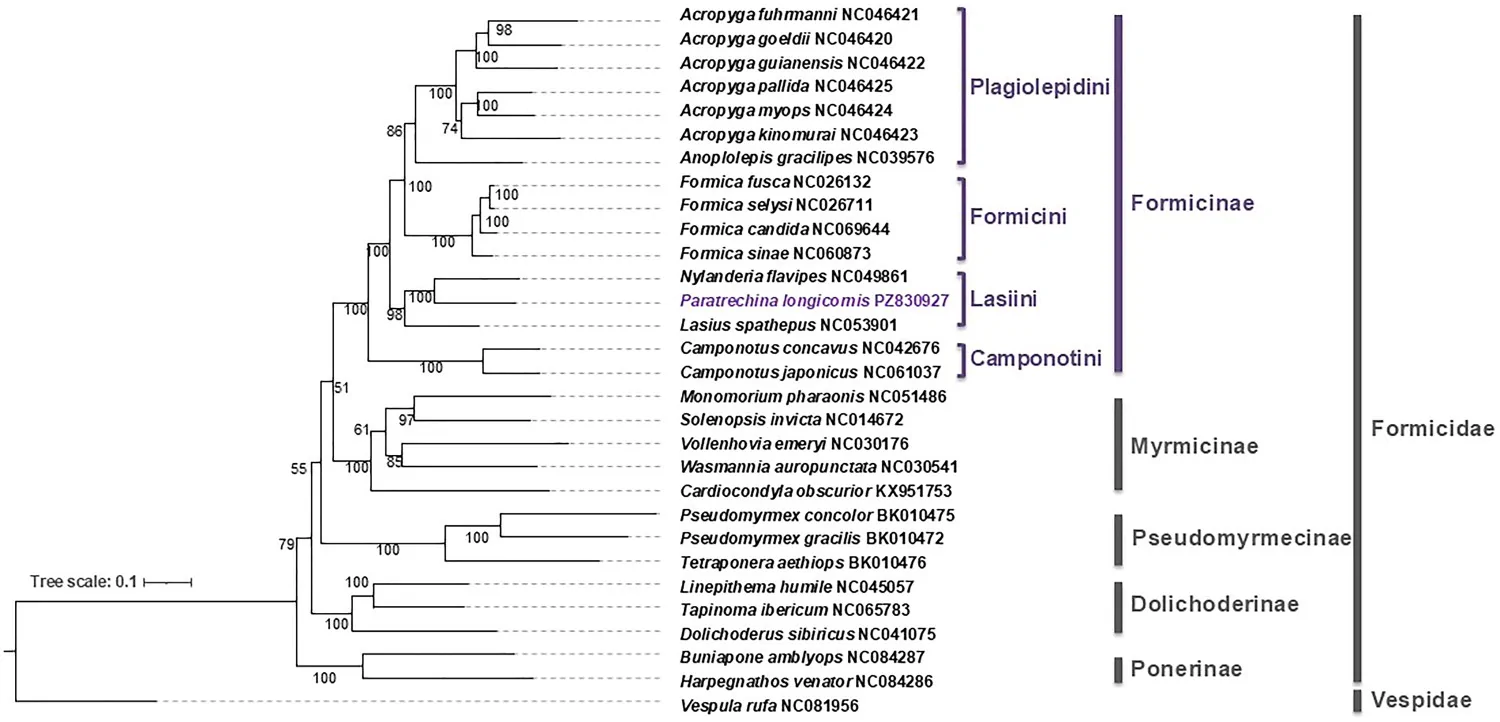
Phylogenetic relationships among 30 Hymenoptera (Formicidae and Vespidae) species based on the nucleotide sequences of 13 PCGs in the mitogenome. *Vespula rufa* was used as an outgroup. Phylogenetic trees were constructed using maximum likelihood (ML) methods. Numbers on nodes indicate ML bootstrap values (%). The GenBank accession numbers were marked after the species name. The following sequences were used: *Acropyga fuhrmanni* (NC046421), *Acropyga goeldii* (NC046420), *Acropyga guianensis* (NC046422), *Acropyga pallida* (NC046425), *Acropyga myops* (NC046424), *Acropyga kinomurai* (NC046423), *Anoplolepis gracilipes* (NC039576; Lee et al. 2018), *Formica fusca* (NC026132; Babbucci et al. 2014), *Formica selysi* (NC026711; Yang et al. 2016), *Formica candida* (NC069644; Liu et al. 2024), *Formica sinae* (NC060873; Zhang et al. 2022), *Nylanderia flavipes* (NC049861; Park et al. 2020), *Paratrechina longicornis* (PZ830927; this study), *Lasius spathepus* (NC053901; Park and Park 2021), *Camponotus concavus* (NC042676; Park et al. 2019a), *Camponotus japonicus* (NC061037; Shen and Li 2022), *Monomorium pharaonis* (NC051486), *Solenopsis invicta* (NC014672; Gotzek et al. 2010), *Vollenhovia emeryi* (NC030176; Liu et al. 2016), *Wasmannia auropunctata* (NC030541; Duan et al. 2016), *Cardiocondyla obscurior* (KX951753; Liu et al. 2019), *Pseudomyrmex concolor* (BK010475; Vieira and Prosdocimi 2019), *Pseudomyrmex gracilis* (BK010472; Vieira and Prosdocimi 2019), *Tetraponera aethiops* (BK010476; Vieira and Prosdocimi 2019), *Linepithema humile* (NC045057; Duan et al. 2016), *Tapinoma ibericum* (NC065783; Ruiz-Mena et al. 2022), *Dolichoderus sibiricus* (NC041075; Park et al. 2019b), *Buniapone amblyops* (NC084287; Xiong et al. 2023), *Harpegnathos venator* (NC084286; Xiong et al. 2023), and *Vespula rufa* (NC081956; Euo et al. 2024).

## Discussion and conclusions

The complete mitogenome of *P. longicornis* reported here is expected to provide basic data for studies investigating population history, molecular systematics, and phylogeography, and also contribute to a better understanding of the mitochondrial rearrangements that occurred during Formicidae evolution. The phylogenetic position recovered in this study is generally consistent with previous classifications based on morphological characteristics and molecular markers, supporting the current placement of *P. longicornis* within Formicinae (Ward et al. 2016; Boudinot et al. 2022). Any discrepancies between mitochondrial genome-based phylogenies and previous analyses may reflect differences in taxon sampling, analytical methods, evolutionary rates among mitochondrial genes, or the maternal inheritance pattern of mitochondrial DNA. In particular, highly invasive species like *P. longicornis* represents an extraordinary model system due to its remarkable diversity and ecological dominance, highlighting its sustained relevance in scientific research. Recent advances in sequencing technologies have facilitated detailed analyses of ant mitochondrial DNA, enabling more comprehensive insights into mitochondrial architecture and function in ants. Further analyses incorporating additional ant species to investigate its evolutionary relationship through the mitochondrial genome provide a powerful framework for disentangling the complex patterns of variation and adaptation that characterize this species.

## Supplementary Material

Supplemental Material

## Data Availability

The genome sequence data supporting the findings of this study are publicly available under GenBank accession no. PZ830927. The associated BioProject, SRA, and Bio-Sample numbers are PRJNA1512077, SRR40145616 and SAMN62375650, respectively.
